# Pacing Forward in the Face of Fragility: Lessons From African Institutions and Governments' Response to Public Health Emergencies

**DOI:** 10.3389/fpubh.2021.714812

**Published:** 2021-11-25

**Authors:** Byron Bitanihirwe, Derrick Ssewanyana, Ismael Ddumba-Nyanzi

**Affiliations:** ^1^Humanitarian and Conflict Response Institute, University of Manchester, Manchester, United Kingdom; ^2^Alliance for Health Development, Lunenfeld-Tanenbaum Research Institute, Toronto, ON, Canada; ^3^Palladium Group LLC, Kampala, Uganda

**Keywords:** Africa, COVID-19, Ebola, education, locust swarms, innovation, public health policies, public health emergencies

## Abstract

Africa is home to 54 United Nation member states, each possessing a wealth of ethno-cultural, physiographic, and economic diversity. While Africa is credited as having the youngest population in the world, it also exhibits a unique set of “unfortunate realties” ranging from famine and poverty to volatile politics, conflicts, and diseases. These unfortunate realities all converge around social inequalities in health, that are compounded by fragile healthcare systems and a lack of political will by the continent's leaders to improve smart investment and infrastructure planning for the benefit of its people. Noteworthy are the disparities in responsive approaches to crises and emergencies that exist across African governments and institutions. In this context, the present article draws attention to 3 distinct public health emergencies (PHEs) that have occurred in Africa since 2010. We focus on the 2013–2016 Ebola outbreak in Western Africa, the ongoing COVID-19 pandemic which continues to spread throughout the continent, and the destructive locust swarms that ravaged crops across East Africa in 2020. Our aim is to provide an integrated perspective on how governments and institutions handled these PHEs and how scientific and technological innovation, along with educational response played a role in the decision-making process. We conclude by touching on public health policies and strategies to address the development of sustainable health care systems with the potential to improve the health and well-being of the African people.

## Introduction

The evidence is clear that public health emergencies (PHEs) can dramatically impact the substantial gains made in primary health care initiatives (1), with estimates suggesting that each year one out of five World Health Organization (WHO) member states experiences a PHE (2).

According to the Model State Emergency Health Powers Act, a PHE can be defined as: “*an occurrence or imminent threat of an illness or health condition, caused by bioterrorism, epidemic or pandemic disease, or novel and highly fatal infectious agent or biological toxins, that poses a* substantial risk of a significant number *of human fatalities or incidents of permanent or long-term disability*” (3). The effects of PHEs are further exacerbated within continents that are comprised of fragile states—such as Africa—with inadequate health care systems (4).

The African continent exhibits a unique set of characteristics including a large ethnic diversity (5), distinct physiographic patterns (6), vast mineral wealth (7) and a burgeoning youth population (almost 60% of the continent are aged below 25) (8). While intriguing, several countries across the continent continue to experience complex emergencies and health crises ranging from civil conflict and infectious diseases (e.g., HIV/AIDS and malaria) to severe drought and malnutrition. These humanitarian crises place significant strain on personal lives leading to socio-economic instability, forced migration, and long-term refugee problems (9, 10), that in turn have an adverse effect on attainment of the United Nations (UN) Sustainable Development Goals.

Given the inadequate funding to PHE and disaster preparedness in many African countries (11), there remains some degree of uncertainty regarding the ability of the continent's countries to adequately respond to these concerns (12)—although the last decade has seen a significant increase in funding for research capacity in Africa (13). Due to the unpredictable nature of PHEs, it is perhaps unsurprising that PHE preparedness (PHEP) is an inherently complex process that involves a range of prevention, mitigation, and recovery activities that extend beyond just enabling a response to emergencies (14). Notably, PHEP is conceptualized as comprising 3 broad elements including: (1) pre-planned and coordinated rapid-response capability; (2) strengthening expertise and building a fully staffed workforce; and (3) ensuring accountability and quality improvement (14). Here we draw attention to African governments and institutions in relation to the handling of 3 distinct PHEs—namely, Ebola, COVID-19, and locust swarms—and how scientific and sustainable technology implementation, along with other effective and innovative responses, have played a role in their managing of these humanitarian crises.

## Ebola Outbreak in West-Africa

Of the 34 documented Ebola outbreaks that have occurred since the first description of the virus in the Democratic Republic of Congo (DRC) in 1976 (15), the 2013–2016 West Africa epidemic was the largest and most widespread in history—culminating in more than 28,000 cases and over 11,000 deaths (16). Epidemiologists identified the index case of the outbreak in Meliandou, Guinea, and fruit bats are believed to have served as a reservoir of the virus and to be involved in the zoonotic spill-over effect that led to a cascade of contagion that spurred the high number of Ebola cases and case fatality rate (CFR) in West Africa (15, 17).

An estimated US$ 2.2 billion in gross domestic product (GDP) was lost in 2015 by the 3 most affected countries (Liberia, Sierra Leona, and Guinea) (18). The epidemic also resulted in lower investment and a substantial loss in private sector growth, declining agricultural production that led to concerns about food security, and a decrease in cross-border trade as restrictions on movement of goods and services increased (19).

Another consequence of the virus relates to rural-urban gradients of transmission and population–level beliefs and practices (i.e., shifts in where care was sought) (20–22), with a pervasive stigmatization of survivors playing a significant role (23). The stigma attached to Ebola has been reported to have led to social inequalities and mental health problems with a large portion of individuals afflicted by the disease suffering hostility and economic hardship (24, 25).

The Ebola outbreak in West Africa also highlighted various barriers to coordinated rapid-response capacity and the need for more robust global health security, particularly in settings with limited public health capacity (16, 26). Indeed, the 3 countries most affected by the outbreak exhibited similar characteristics including: inadequate financial resources and health care systems (as reported by low numbers of nurses and doctors) in addition to a scarcity of medicines and personal protective equipment (PPE)—each of which represent a unique threat to containing the spread of infectious disease, and in turn provide a hurdle toward implementation of the International Health Regulations (IHR) (27). Notably, porous borders meant that the Ebola outbreak was not just restricted to Guinea, Liberia and Sierra Leone as cases were also reported in Nigeria, Senegal, and Mali (15, 28). The slow recognition and delayed response to control the Ebola outbreak by West African governments, exposed defective containment strategies and poor crisis management in the countries' worst affected by the virus. Inadequate contact tracing and detection of suspected cases coupled with poor surveillance and gaps in the community's knowledge about the Ebola virus contributed to a rampant spread of the disease (29). Unfortunately, during the outbreak, governments in Liberia, Guinea and Sierra Leone failed to communicate effectively with citizens. In Liberia, this resulted in frustrations and riots in the capital city, Monrovia.

### Improving Pre-planned and Coordinated Rapid-Response Capacity

As the effects of the Ebola virus continued to unfold in West Africa a key strategy shown to moderate the crippling effects of the epidemic involved an integrated and calibrated response strategy that included: (1) bolstering standardized supportive care of survivors via treatment for the symptoms and complications of Ebola (e.g., mental health and psychosocial support), (2) leveraging and deploying aid through international organizations such as the WHO and Médecins Sans Frontières; (3) funding for emergency Ebola treatment; (4) rapid and accurate Ebola diagnostics testing through platforms such as real-time polymerase chain reaction; (5) scaling-up of national disease surveillance activities (e.g., digital health/apps via mobile devices); (6) a licensed Ebola vaccine (Merck's VSV-ZEBOV vaccine) and (7) focusing on social science and community engagement through aspects of risk perception, tackling vaccine hesitancy and education as a means to minimize confusion and to empower individuals to adopt preventative behavior (30). This response strategy is estimated to have taken over a year to implement (31, 32), with follow-up visits provided to each survivor every month over a period of 6 months and then every 3 months for a year (30).

Six years on from the beginning of the Western Africa Ebola epidemic, the DRC has been grappling with its 12th Ebola outbreak. Active conflict, a severe measles outbreak and insecurity make this epidemic one of the most complex ever encountered (33). However, the Ministry of Health of the DRC has mounted an impressive response strategy to the outbreak with international support from the United States Centers for Disease Control and Prevention (CDC), WHO and Gavi, the vaccine alliance. Beyond placing an emphasis on developing local health care systems in the most affected areas (i.e., Kivu and Ituri), a strong aspect of the DRC Ebola response has been applying lessons learned from the outbreak in West Africa (30). These lessons include community engagement, better support of survivors, use of mobile phone data to inform the dynamics of Ebola transmission (via travel patterns and contact tracing) (34), and licensed approval for two vaccines (Merck's single dose VSV-ZEBOV vaccine and Jansen's two-dose vaccine regimen of Zabdeno and Mvabea), with recent estimates suggesting that more than 300,000 people have been immunized against Ebola through vaccination in the DRC (35). Despite the significance of the current vaccines against Ebola, there still remains the need to develop an effective strategy for optimal impact of vaccination (36). In this respect, Coltart et al. emphasize that prophylactic vaccination of health care workers (HCWs) could have a substantial epidemic-reducing effect on the spread of Ebola (37). Other evidence from mathematical and statistical models suggests that engaging HCWs to deliver vaccinations represents both a feasible and effective strategy that may be implemented in a future Ebola outbreak (38, 39).

### Building Expertise and Fully Staffed Workforce

Several notable initiatives have been developed to enhance capacity (viz., expertise) and build leadership within the local workforce at national and local levels in various countries, as a means of strengthening sustainable PHEP. For instance, the CDC's Surveillance Training for Ebola Preparedness (STEP) initiative was shown to be a successful mentorship and competence-based initiative that collaborated with various local training institutes and organizations to rapidly build the surveillance capacity of district surveillance officers in Mali, Guinea-Bissau, Senegal and Ivory Coast during the Ebola outbreak in West Africa (40). Along with the STEP initiative, implementation of laboratory capacity building programs to strengthen bio-risk and quality management systems, diagnostics and facility engineering, and bio-surveillance capacity was able to bolster emergency preparedness and response (41). Perhaps most notably, infection prevention and control capacity building programs for HCWs registered positive benefits on knowledge and practices of HCWs in the fight against Ebola outbreak in DRC (42) and Western Africa (43). Also, foreign medical worker deployment from the African Union Support to the Ebola Outbreak in West Africa and medical personnel from Cuba played a central role to fill the gap for skilled HCWs, as well as co-learning for skills development (44).

### Accountability and Quality Improvement

In West Africa, community monitoring—which involves providing patients with information and enabling a public forum to monitor frontline workers—was beneficial in generating some form of social accountability and trust. An example of an effective community monitoring program was the Liberian government's door-to-door canvassing campaign during the Ebola epidemic (45). Through the Financial Tracking Service (FTS) of the United Nations Office for the Coordination of Humanitarian Affairs (UNOCHA), curated financial data (e.g., funding needs, commitments, pledges and projected funding) on Ebola virus outbreak were continuously updated and accessible in downloadable format on the UNOCHA website to facilitate accountability and transparency (46).

## COVID-19 Outbreak in Africa

The first case of coronavirus disease 2019 (COVID-19), the disease caused by severe acute respiratory syndrome coronavirus 2 (SARS-CoV-2), was reported on the African continent on February 14, 2020 in Egypt, with Sub-Saharan Africa (SSA) detecting its first case in Nigeria on February 27, 2020 (47). In most countries, the initial response to the COVID-19 pandemic was strong and proactive.

Despite these measures, many public health experts predicted that the pandemic would severely overwhelm Africa's largely fragile and underfunded health systems. Of the 34 African countries surveyed in the WHO COVID-19 readiness status report, only 10 countries reported adequate capacity to respond to the epidemic, including with PPE for the population (48). The UN Economic Commission for Africa estimated that, in the worst-case scenario, 3.3 million Africans would die from the disease (49). Concerns over the combination of overstretched, underfunded health systems and the existing load of infectious and non-infectious diseases often led to scenarios being talked about in apocalyptic terms.

More than a year into the pandemic, the continent has however thwarted most predictions regarding the spread of the virus. The health and social measures to contain the COVID-19 epidemic implemented by most countries are likely to have slowed the spread of the virus, and the number of confirmed cases and deaths in Africa remained lower than initially forecast. As of October 18, 2021, confirmed cases of COVID-19 from 55 African countries reached 8.4 million with a CFR of 2.6% (i.e., 215,784 deaths) (50). By early August 2021, it was estimated that only 3.5% of the global COVID-19 cases and 4.1% of the global COVID-19 related deaths were from Africa (50, 51)—a continent that accounts for 17% of the global population (52).

Nevertheless, the magnitude of the challenge and the continent's underlying vulnerabilities should never be underestimated. The weak PHE management systems in most countries, have rendered it difficult to discern accurate transmission, hospitalization and mortality rates (53). For example, currently, the continent's testing rate is one of the lowest in the world. Therefore, the full scope of the pandemic remains uncertain. In addition, several countries are experiencing a second wave of the pandemic and some, such as Kenya, Egypt and Tunisia, have seen a third wave (54). This new wave of infections is thought to be associated with the emergence of variants that are more transmissible. Unfortunately, only a few countries have the capacity to carry out the specialized genomic sequencing required to detect coronavirus variants.

Further, the health and economic shocks occasioned by the pandemic threaten to wipe out decades of economic progress and development gains in Africa. These risks put some countries on an unsustainable debt path (55). The pandemic has also laid bare structural shortcomings such as inadequate health, educational and technological infrastructure, limited social protection, gender inequality, large informal economies, lack of access to basic services, and constrained fiscal policy space (56). For example, the contraction in per capita GDP growth caused by the pandemic may have pushed an additional 26.2 million to 40 million (i.e., 2–3%) people into extreme poverty in SSA by the end of 2020 (57). Fighting a pandemic and its economic aftershocks requires enormous amounts of money. In higher income countries, governments have stepped forward with trillions in economic stimulus packages. But most developing countries do not have the money to cover the full costs of this pandemic.

### Improving Pre-planned and Coordinated Rapid-Response Capacity

To forestall the COVID-19 health and economic crisis, most African countries developed response plans. Specifically, most African governments rapidly implemented public health and social measures to contain the pandemic, including closing borders, mandatory general lockdown, physical distancing measures, and establishing centers for quarantining of cases (58). Furthermore, response plans have also majorly centered around four main areas simultaneously, including: (1) saving lives; (2) protecting poor and vulnerable citizens and responding to the impact on their livelihood; (3) protecting and creating jobs through support to private sector and (4) building back better systems (59). For example, to save lives, several African countries focused on intensive surveillance and case-finding, leveraging the Integrated Disease Surveillance and Response framework (IDSR) (60). The Partnership to Accelerate COVID-19 Testing (PACT) Initiative, for instance, is an initiative launched by the African Union Commission and the Africa CDC to boost and coordinate procurement and supply chains for medical supplies and to support protracted testing for COVID-19 within the African setting (61). The continent has also been able to draw on previous experience in dealing with PHEs such as the Ebola crisis to make better decisions on public health and social measures. For example, several countries focused their response efforts toward community engagement, risk communication, and locally adapted innovations in tracing, treatment and isolation (56). With the vaccine roll-out underway in many African countries, ensuring an adequate supply of vaccines is a priority for the region. Countries have mainly accessed vaccines through the COVAX Facility, bilateral deals, and donations. Nonetheless, concerns regarding disparities in vaccine access and distribution remain widespread (62). Many developed countries have displayed a very high degree of “vaccine nationalism,” locking up most supplies and prioritizing the vaccination of their entire populations before releasing surpluses to protect even the most vulnerable populations in low- and middle-income countries (LAMICs) (63). As of October 16, 2021, Africa had administered 12.5 doses of COVID-19 vaccines per 100 people. The vaccination rate of the continent was far slower than the world average measured at 84.5 vaccines per 100 individuals on the same date (64). Further, while concerted global efforts are working to accelerate equitable access, vaccine hesitancy—driven in part by a trust deficit between communities and the actors leading vaccine rollout—risks prolonging the pandemic and its secondary waves of conflict and economic devastation.

### Building Expertise and Fully Staffed Workforce

Across the African continent, HCWs are boosting their emergency response skills in tackling COVID-19, for example through virtual and in-person trainings organized by Ministries of Health and health organizations or research institutions (65). The PACT initiative for example, also focuses on the support for training and deployment of one million community HCWs to support contact tracing within the African setting (61). Government Ministries of Health have learned to harmonize research activities, through leveraging research laboratory capacity (both personnel and equipment) of academic research institutions and other in-country laboratories for community COVID-19 testing (66); as well as building foreign/international research partnership to improve testing or medical product development capacity (67). It follows that efforts to build research capacity to conduct good quality collaborative international COVID-19 vaccine trials in Africa will allow for better protection against this devastating infectious disease (68, 69).

### Accountability and Quality Improvement

With regard to economic response, Africa's fiscal realities limit what most countries can do to alleviate pressures on citizens (56). Several countries have undertaken measures to address the economic fallout of the pandemic. For example, some countries announced remedial fiscal and monetary measures, as well as food distribution and financial support to the most vulnerable groups. However, less has been done across countries to cushion against lost income and export earnings, dwindling remittances, and decreased government revenue. In addition, relatively few countries have articulated initiatives to mitigate the socio-economic impacts of COVID-19 in the long-term. Therefore, the road to recovery will be long and vary significantly across countries (48). Most African countries continue to rely on foreign aid in response to the impact of the pandemic. Since the start of the pandemic in March 2020, the World Bank has made available nearly US$ 24.7 billion to respond to the COVID-19 crisis through a combination of new operations in health, social protection, economic stimulus and other sectors, as well as redeployment of existing resources (70). Several African countries have also received foreign assistance from bilateral partners to help prevent, detect, and respond to the COVID-19 pandemic and strengthen their public health preparedness. For instance, France mobilized €1.2 billion to fight the spread of COVID-19 in the most vulnerable countries, most of which are in Africa (71). As with Ebola, the FTS of the UNOCHA has curated financial data on COVID-19 emergency funds as part of the Global Humanitarian Response Plan that are continuously updated and accessible through the UNOCHA website (72). Initiatives, for example by the African Union, have enabled increased dialogue and opportunity for learning and sharing among government officials, audit institutions, procurement oversight bodies, and civil society organizations on the African continent in relation to innovative accountability mechanisms and crisis budget support operations (73).

## Locust Swarms in East-Africa

The impacts of the PHEs presented above are devastating enough without additional social and economic dislocation caused by non-disease outbreaks. In 2020, East Africa faced just such a situation when a surge of desert locusts invaded the Horn of Africa. In order to fast-track effective response toward the attack, on January 17, 2020, the UN's highest level of emergency (L3 protocols) was activated by the Director-General of the Food and Agriculture Organization (FAO) (74). Beyond widespread hatching, band and swarm formation in north East Ethiopia (57,450 hectares had been treated), immature swarms prevailed in Somalia (17,477 hectares) and to a lesser extent in North West Kenya (2100 hectares) (75). Desert locust infestation was also reported in 24 districts in Uganda around a similar timeframe (76). In Kenya, the 2020 desert locust invasion is considered the worst in 70 years (77).

The combination of the ongoing COVID-19 pandemic and a desert locust outbreak has exerted an enormous economic toll and an even greater burden on the health systems in East Africa. The meager financial resources, which would have been fully vested into the COVID-19 programmes within East African governments, had to be rationed so that some resources are used to combat desert locusts, and this called for more borrowing. For instance, more than US$ 160 million was loaned to the East African countries of Kenya, Uganda, Djibouti and Ethiopia by the World Bank, to combat desert locusts (78) and yet additional loans for COVID-19 had also been secured by some of these governments [for instance US$ 1 billion for Kenya (79) and more than US$ 15 million for Uganda (80)] which further deepens their debt crisis.

### Improving Pre-planned and Coordinated Rapid-Response Capacity

Through support from the World Bank, national response programs, i.e., Uganda Emergency Desert Locust Response Project (81), Kenya emergency locust response program (82) and Ethiopia Emergency Locust Response Project (83) were set up in early 2020. As part of the commitment plans, actions were stipulated through which desert locust control programs would be implemented in accordance with social and environmental standards, for example: environmental and social assessment of risks arising from the projects, occupational health and safety measures, and pollution prevention and management strategies (81–83). These programs also were set up with in-country coordination plans. For instance, in Kenya, a multi-institutional technical team on desert locusts was established to coordinate policy and technical advisory on desert locust management which was tasked with activities like providing advisory to county administrations and any other stakeholders, planning the collection and collation of technical information and building capacity among stakeholders on integrated desert locust management (77). Surveys of terrain, state of habitat and locust population were performed so as to inform policy and decision making (83). Strengthening of existing systems to combat future outbreaks, for example the Locust Control Unit within the Plant Protection Service Division of Kenya's Ministry of Agriculture, was among the strategic aims of the funding from the World Bank (77). FAO encouraged country-level partners to record and transmit desert locust related surveillance data to ministerial organizations (like the Ministries of Agriculture) so that such essential information is included and utilized in FAO's Desert Locust Information Service (DLIS). In each country, a Locust Information Officer is responsible for collating, analyzing and transmitting this data to DLIS (84). In turn, the DLIS analyses the data and keeps countries informed of the current situation and expected developments by providing a forecast up to 6 weeks in advance (84). Data sharing for improved monitoring of desert locusts is also being boosted through mobile-phone based surveillance technology such as the eLocust3m and other platforms like the centralized Desert Hub platform (74). According to FAO, the primary method for controlling the 2020 desert locust swarm and hopper bands, is through organophosphate chemicals, delivered by vehicle mounted aerial sprayers and knapsack or hand-held sprayers (85). The main strategy involves targeting breeding grounds and controlling the hopper bands while still at the nymph stage—that is before they can fly (77). More recently, test drones equipped with mapping sensors and atomizers have been deployed to spray pesticides to tackle the desert locust swarms in East Africa. Governments and donor agencies (e.g., FAO and the World Bank) have ensured that there is disaster recovery relief provided, including inputs such as seed-fertilizer and pesticides to selected farmers faced with hardship and also provided fodder seed to affected communities to restore lost pastures, emergency food security mechanisms and direct cash transfers (76, 83, 86). In Uganda, for example, as part of World Bank's US$ 48 million loan, funds were set aside to boost existing savings and investment platforms/groups at village level through a Village Revolving Fund and seasonal income transfers to vulnerable (76).

### Building Expertise and Fully Staffed Workforce

Capacity building of in-country human resource was conducted. For example, the FAO facilitated training of National Youth Service trainees as part of boosting the Government of Kenya ground surveillance for desert locusts (85). Governments also mobilized and trained communities to establish locust surveillance systems based at community, district and national levels so as to ensure the sustainability of mapping monitoring and surveillance (76). By the start of the desert locust disaster, FAO was already coordinating with over 100 NGOs in Ethiopia in using and building capacity for the use of eLocust3m (86).

### Accountability and Quality Improvement

The FTS of the UNOCHA has played an integral role in enabling timely access to financial data on humanitarian funding flows on desert locust response across East Africa and the Horn of Africa (87). As part of quality improvement—in terms of the potential unintended negative consequences of pesticide use—initiatives were put in place to monitor and assess the environmental and human health risks attributable to their use (76, 77).

## Implications for Policy and Research

Evidence-informed policy and decision-making is crucial for ethical and sustainable response to PHEs. The generation and translation of evidence to inform policy and decision-making is often seen as a race against time—with the quality, depth and conciseness of available evidence directly affecting policy decision-making processes (88). Notable in this regard are rapid assessment tools, developed as a public health approach to speed up—and bring together—the processes of evidence-based decision-making during crisis management of PHEs (89, 90). The present paper emphasizes the necessity of utilizing the rapid assessment approach in a more collaborative and engaging manner as a means of facilitating dialogue between decision-makers and other stakeholders (including scientists and communities) in relation to programme planning and interventions which, in turn, enable PHEP and responses “*in-the-now*” (91). Indeed, rapid assessment tools have been applied successfully around the world to generate evidence for decision-making in the management of a variety of PHEs, including HIV/AIDS (92), forced displacement due to conflict (93), natural disasters (90) and more recently COVID-19 (94). Such tools are vital for identifying and addressing context specific issues, in acting as a guide for resource allocation and providing key information in relation to response planning and implementation as evidenced during the 2013–2016 Ebola epidemic in West Africa (95).

Each of the PHEs described in our paper has important lessons. Notably, comprehensive and reliable data generated through well-designed and well-executed research (e.g., real-time epidemic forecasting and disease surveillance through administrative data systems) will prove important in resolving important research questions and existing knowledge gaps. It follows that any research during PHEs should only be conducted if it has high social value (i.e., it provides information to support the immediate response, either through evidence to assist the decision-making process or targeted interventions aimed at minimizing the magnitude of the harm suffered by a population) (96). Importantly, the knowledge generated through research in anticipation of, during, and after a PHE is vital to building future capacity to better achieve the goals of preparedness and response: preventing illness, injury, disability, and death and supporting recovery (97). However, conducting research in PHE settings often presents a number of challenges, including an inability to access affected people, insecure settings and lack of research infrastructure (e.g., underdeveloped oversight and regulatory bodies of host countries) (98–100).

In recent years digital health technologies have been harnessed as a means of data collection in a variety of research settings including PHEs (101). Specifically, these technologies improve the quality and efficiency of research studies via automated data capture and by improved data traceability, reliability and provenance (102). Additionally, digital technologies can improve study transparency, security, informed consent, handling of confidential patient information and data sharing (102). This stated, challenges exist in relation to data sharing mechanisms as well as the technical and legal ability to protect intellectual property (e.g., inventions/innovation and research publications), particularly in the context of LAMICs.

Some solutions toward improving research in the PHE context include appointing a coordinator for scientific research—a role that involves coordinating the research process, identifying mechanisms and rapid funding schemes to support research, enlisting existing research networks in order to coordinate and accelerate research efforts (e.g., for data collection), and establishing a centralized institutional review board to provide timely reviews of multiagency studies (97).

In line with the lessons learned from our paper, Khan and colleagues (103) identified a number of important considerations/factors for enhancing research, policy and practice related to preparedness and response to PHEs which include governance and leadership, community engagement, risk analysis, surveillance and monitoring, resources, investment in systems strengthening and capacity building, communication, research and learning, and evaluation (103). Each of these factors are described in further detail—in relation to their significance in policy decision-making processes—in Table 1.

**Table 1:** Summary of supportive factors for improved policy and practice for public health emergency preparedness and response.

**Factor**	**Description**
Governance and leadership	° Ensures high standards of vertical and horizontal integration of organization structures, partnerships, and accountabilities to support coordinated and interoperable functioning ° Promotes clarity, defines and delineates roles and enables organizational flexibility
Community engagement	° Provides inclusive support and proactively seeks out community values, concerns, and aspirations ° Enables consideration of community assets and values in addition to facilitating transparency and trust
Risk analysis	° Represents a means to understand risks for the community and to better access and analyse information ° Facilitates informed decision-making and planning
Surveillance and monitoring	° Integral to developing robust surveillance, data collection/analysis, and information processes to build a shared understanding between key stakeholders and the community ° Facilitates awareness in advance and analysis of impacts of public health actions to guide response planning
Resources	° Scalability of resources and sufficient physical infrastructure can promote adaptive capacity and support decision-making ° Important to establishing resource mobilization priorities in relation to the allocation of limited resources
Investment in systems strengthening and capacity building	° Expanding and developing the base of well-trained and knowledgeable people as a means for bolstering health care infrastructure and interoperability ° Focusing on multi-pronged initiatives (e.g., funding and human-centered design)
Communication	° Sharing of information in a way that is understandable in terms of raising awareness ° Allows for feedback and to engage with diverse audiences when supported by sufficient capacity
Learning and evaluation	° Assessments are integral to recovery and building back better and more successful if prioritized and delivered in a timely manner ° Fosters improvement and change for better PHE preparedness and response ° Strengthening multidisciplinary and multisectoral learning and collaboration with clear planning for disaster risk management/reduction
Research	° Developing research on the impact of adversity, resilience and disaster recovery mechanisms in PHE settings (including the African context) ° Bolstering the investment in research and local innovation for improving response mechanisms to better maximize resources in a contextually appropriate manner

## Conclusions

Public health emergencies in the African region continue to exert an enormous toll on people's livelihoods—with some PHEs characterized by excessive mortality and morbidity rates—often testing collective resilience. Unfortunately, there are significant challenges that surround coordinated rapid response capacity, staffing and capacity building, and quality improvement with respect to most of the PHE response efforts on the African continent, which points to systemic fragility. Compounding matters further, PHEs and some of their secondary effects are bi-directional, and these secondary effects—such as distrust of health authorities and disruption to health services—are seen to make it harder to combat these humanitarian crises (104, 105).

Notwithstanding, there have been vital lessons learned from previous PHEs and African governments, institutions and partners have devised various initiatives toward more holistic PHEP. In light of the PHEs discussed in this paper, namely Ebola, COVID-19 and locust swarms, initiatives to strengthen pre-planned and coordinated response have included: containment measures (e.g., social distancing and border restrictions); building local and international collaborations to leverage expertise, international aid, and other resources; scaling-up surveillance and monitoring activities; leveraging initiatives like COVAX to ensure vaccine roll-out and supply; management and treatment of survivors; social protection programs against shocks to livelihoods; and community engagement. To develop expertise and a well-staffed workforce, various training programs as part of capacity building (e.g., in surveillance, laboratory work, infection prevention and control, data management) coupled with mentorship and leadership training have been found to be beneficial. Foreign worker deployment has also been critical especially in relation to the Ebola virus outbreak. Lastly, as a means of improving accountability and quality improvement in PHEP, African governments and institutions have utilized the Financial Tracking Service of the UNOCHA to monitor humanitarian financial assistance and commitments. Dialogue between governments and institutions on innovative accountability mechanisms and crisis budget support operations has strengthened cross-learning on best practices for accountability. Some countries have also devised community monitoring approaches to improve trust and monitor quality of services during PHEs. Overall, adoption of system-wide approaches matched by scaling-up innovations to achieve impact may prove effective to better deal with the negative outcomes related to complex PHEs on the African continent. There will also be a need to refine policies on leadership relating to PHEP and response in conjunction with policies that focus on strengthening national and technical capacities that align with the IHR as a means of accelerating progress toward universal health coverage.

## Data Availability Statement

The original contributions presented in the study are included in the article/supplementary material, further inquiries can be directed to the corresponding authors.

## Author Contributions

All of the authors substantially contributed to the conception and drafting of this manuscript.

## Conflict of Interest

The authors declare that the research was conducted in the absence of any commercial or financial relationships that could be construed as a potential conflict of interest.

## Publisher's Note

All claims expressed in this article are solely those of the authors and do not necessarily represent those of their affiliated organizations, or those of the publisher, the editors and the reviewers. Any product that may be evaluated in this article, or claim that may be made by its manufacturer, is not guaranteed or endorsed by the publisher.
